# Benchmarking of the BITalino biomedical toolkit against an established gold standard

**DOI:** 10.1049/htl.2018.5037

**Published:** 2019-03-21

**Authors:** Diana Batista, Hugo Plácido da Silva, Ana Fred, Carlos Moreira, Margarida Reis, Hugo Alexandre Ferreira

**Affiliations:** 1 Instituto de Telecomunicações, Instituto Superior Técnico, 1049-001 Lisboa, Portugal; 2 Escola Superior de Tecnologia, Instituto Politécnico de Setúbal, 2910-761 Setúbal, Portugal; 3 Department of Bioengineering, Instituto Superior Técnico, 1049-001 Lisboa, Portugal; 4 Instituto de Biofísica e Engenharia Biomédica, Faculdade de Ciências da Universidade de Lisboa, 1649-004 Lisboa, Portugal

**Keywords:** physiology, feature extraction, electroencephalography, medical signal processing, electromyography, data acquisition, electrocardiography, medical signal detection, mean square error methods, BITalino biomedical toolkit, educational research purposes, BioPac MP35 Student Lab Pro device, methodical experimental protocol, electrodermal activity signals, signal processing techniques, electroencephalography data, physiological signal acquisition, root mean square error, electromyography data, post-processing methods, electrocardiography

## Abstract

The low-cost multimodal platform BITalino is being increasingly used for educational and research purposes. However, there is still a lack of well-structured work comparing data acquired by this toolkit against a reference device, using established experimental protocols. This work intends to fill the said gap by benchmarking the performance of BITalino against the BioPac MP35 Student Lab Pro device. This work followed a methodical experimental protocol to acquire data from the two devices simultaneously. Four physiological signals were acquired: electrocardiography, electromyography, electrodermal activity and electroencephalography. Root mean square error and coefficient of determination were computed to analyse differences between BITalino and BioPac. Electrodermal activity signals were very similar for the two devices, even without applying any major signal processing techniques. For electrocardiography, a simple morphological comparison also revealed high similarity between devices, and this similarity increased after a common segmentation procedure was followed. Regarding electromyography and electroencephalography data, the approach consisted of comparing features extracted using common post-processing methods. The differences between BITalino and BioPac were again small. Overall, the results presented here show a close similarity between data acquired by the BITalino and by the reference device. This is an important validation step for all researchers working with this multimodal platform.

## 1 Introduction

The low-cost biomedical development toolkit BITalino [1, 2] is seeing an increasingly higher use within academia, for educational and research activities in a wide array of application fields [3, 4, 5, 6]. Despite the theoretical characterisation of different components of the system and laboratory benchmarking initially performed [7, 8], a recurring issue that is frequently pointed out regarding its use is the lack of empirical validation against a gold standard device.

Previous work from our group has made an attempt to address this issue, by comparing the BITalino and BITalino (r)evolution toolkits with a biosignalsplux professional biomedical research system [9]. While the findings of this previous study have provided preliminary evidence validating BITalino [10], several methodological and practical aspects have been highlighted to require further evaluation.

In particular, a more formal albeit easily replicable experimental protocol was identified as needed, and the need for benchmarking against a more established and widely used gold standard other than the biosignalsplux was also noted. As such, this Letter has the goal of extending our previous investigation, to provide an ultimate performance assessment of BITalino.

**Fig. 1:**
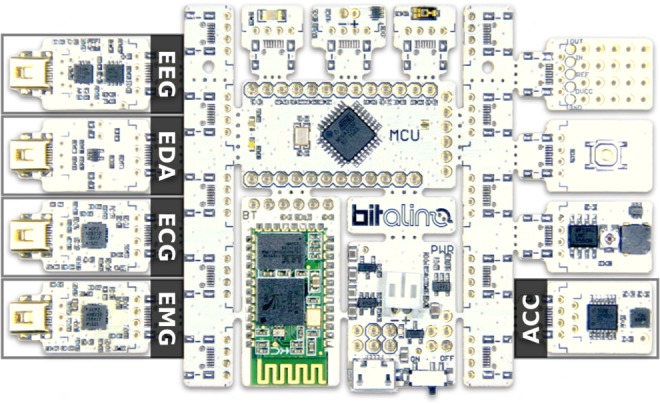
Biomedical sensors bundled by default in BITalino (r)evolution

Within the state-of-the-art, BioPac [11] is perhaps the most well-established and recognised system for biomedical research and education, complete with classroom lessons that define experimental protocols used for decades and comprising biomedical sensors common to those included in BITalino (Fig. 1). In [10], results are already provided regarding the performance of BITalino when compared with its newest counterpart BITalino (r)evolution, thus offering a referential between both, as such, in this Letter we chose to focus only on benchmarking the performance of BITalino (r)evolution versus the BioPac (it is important to highlight that the electroencephalography (EEG) sensor was not available in the first version of BITalino).

The remainder of the Letter is organised as follows: in Section 2, we describe the methodology used in our study; in Section 3, we present and discuss the comparative results; and finally, in Section 4, we outline the main conclusions.

## 2 Materials and methods

### 2.1 Devices overview

Our goal is to benchmark the signal quality of data acquired with the multimodal platform BITalino (r)evolution (Fig. 1) against the more established BioPac MP35 Student Lab Pro (BSL) [11]. Hereinafter, for the sake of simplicity, these devices are referred to as BITalino and BioPac. Additional information about the equipment used during the acquisition is presented in Table 1. We focus in particular on four physiological signals: electrocardiography (ECG), electromyography (EMG), electrodermal activity (EDA) and EEG. Characteristics of these sensors for the BITalino device can be found in the respective datasheets: ECG [12], EMG [13], EDA [14] and EEG [15].

**Table 1:** Sensors’ bandwidths and additional material used with the two devices

Sensor	Bandwidth, Hz		Additional material	
	BioPac MP35	BITalino (r)evolution	BioPac MP35	BITalino (r)evolution
ECG	0.05–35	0.5–40	SS2L electrode lead set	3-lead accessory UC-E6
EMG	5–250	25–480		
EEG	0.5–35	0.8–49		
EDA	0–35	0–2.8	SS3LA EDA transducer	2-lead accessory UC-E6

For data acquisition, we used the OpenSignals (r)evolution software, as it is the recommended software to be used with BITalino, with data being transmitted via Bluetooth (the standard transmission channel for the platform). For the BioPac system, chosen as a gold standard against which the BITalino is being compared, we used the BSL Pro software application, as recommended by the manufacturer. As previously mentioned, BioPac has been chosen due to the widespread use of the BSL package for education and research purposes.

The two devices have different sensors’ bandwidths, which are specified in Table 1. To account for these differences, we will need to do pre-processing with the purpose of conditioning the signals before comparing them, as will be detailed in the next section.

### 2.2 Data acquisition protocol and pre-processing

Data was acquired for seven subjects following the protocol described hereafter. For each sensor, we acquired data simultaneously with the BioPac and the BITalino. Further details about the acquisition process for the four sensors are presented below, in the order in which the acquisition took place for each subject. The protocol followed here is a minor adaptation of the one described in BioPac Student Laboratory Lessons. All data were acquired with a sampling frequency of 1000 Hz and default filtering settings on both devices.

To synchronise the data collected by the two devices while guaranteeing electrical decoupling between them, a light-emitting diode (LED) and light sensor setup were used. The BioPac was programmed to trigger periodic sequential LED pulses, and data from the BITalino light sensor was recorded simultaneously with the biosignal data.

**Fig. 2:**
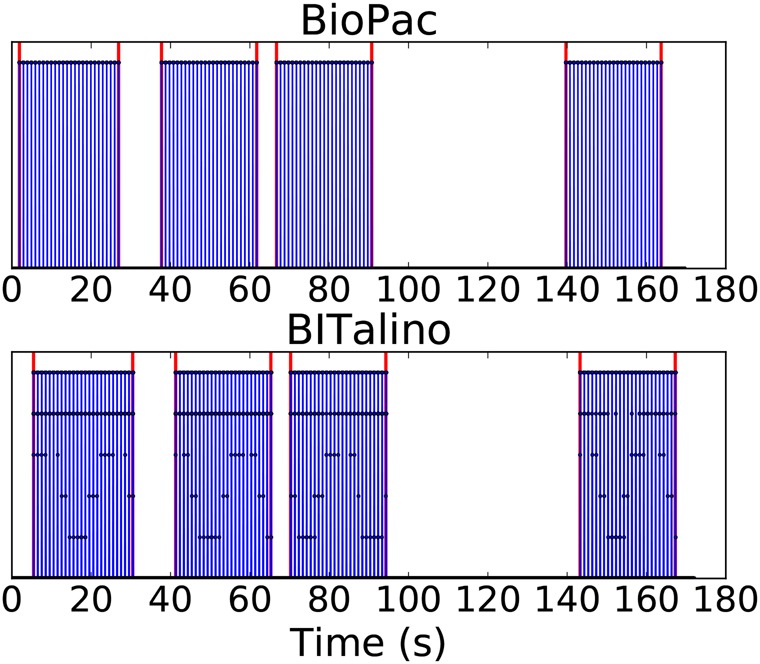
Pulses emitted by the BioPac (top) and acquired BITalino light sensor data (bottom) during an ECG recording, prior to time alignment and synchronisation. Red vertical lines indicate the beginning and end of each one of the four activities

The pulses used were programmed to have a frequency of 1 Hz, width of 100 ms and cover the full scale in amplitude. To help segment the areas of interest in post-processing, sequences of pulses were emitted during ‘activity’ periods and turned off between activities (e.g. when the subjects were changing position). The first pre-processing step consists of segmenting the data by activity periods, and discarding the data acquired between activities. For each subject and each sensor, ‘activity’ periods must be identified and the data should be segmented accordingly. An example of BioPac and BITalino data acquired to allow segmentation is shown in Fig. 2. For each activity, we identify the first and last pulses in the series and detect its onset for both devices. Sensor data is then segmented according to these time stamps.

To have a fairer comparison, all data were filtered prior to this segmentation. A fourth-order forward–backward Butterworth band-pass filter was implemented for each sensor, except for the EDA, where a lowpass filter was used. The cut-off frequencies were chosen according to the most restrictive bandwidths values of Table 1 (e.g. 0.5 and 35 Hz for the ECG; 0.8 and 35 Hz for the EEG).

Further pre-processing steps, specific to each sensor were used for the sake of reinforcing the comparison, and are presented in the following subsections, along with the acquisition protocol.

#### 2.2.1 Electrocardiography

For ECG acquisition, we followed the procedure described in BSL Lesson 5, where three electrodes are used to record the ECG. The ground was placed on the medial surface of the right leg, just above the ankle. A second electrode mimicked this position on the left leg. The third electrode was placed at the wrist, on the right anterior forearm.

The ECG acquisition is divided into four activities of 20 s: supine, seated, deep breathing and recovery after exercise. For the first two activities, the subject is simply asked to relax. During the third activity, he is instructed to take long, slow, deep breaths. After a series of jumping-jacks meant to raise his/her heart rate, the subject is told to sit down and relax again so the fourth activity can be recorded.

For some sensors, including the ECG, there is a slight delay between data from the BioPac and data from the BITalino, which we believe to be introduced by differences in the specification of the hardware signal conditioning circuitry. To achieve the best possible alignment and thus a more correct comparison, the following procedure was followed:

- Data was segmented according to activity periods
- For each segment (activity), the correlation between data from the two devices was computed
- The ‘shift’ was defined as the maximum correlation point such that:
  oThe shift was positive (due to the acquisition protocol, BioPac data was always ahead of BITalino data)
  oThe shift was at most 20 samples (the ‘true’ alignment was always close to the beginning of the segments)
- The BioPac segment starts now after the determined number of ‘shift’ samples

**Fig. 3:**
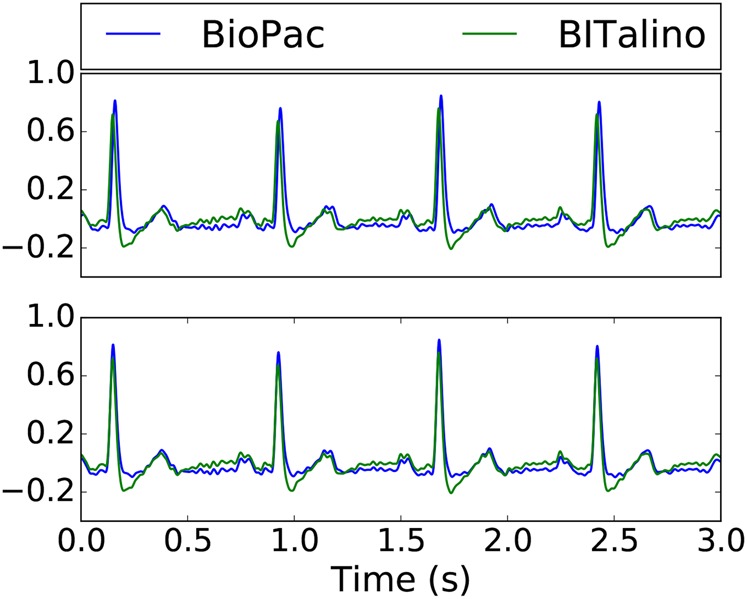
First 3 s of filtered ECG segments before (top) and after (bottom) the alignment procedure. Segments were scaled for visualisation purposes

An example of segments before and after this alignment procedure is shown in Fig. 3 for the ECG.

#### 2.2.2 Electromyography

The EMG data is divided into two activities: clenching fist for the dominant hand and clenching fist for the non-dominant hand. For each of them, one electrode is attached to the anterior forearm, close to the elbow. A second electrode and the ground are placed on the wrist, on the interior side of the arm.

Each activity lasts 1 min during which the subject is asked to clench his/her fist and hold before releasing the clench and repeating the process with an increasingly high clench force. The maximum clench should be achieved on the fourth repetition. The subject repeats this series of four clenches during the 1 min period. This procedure follows what is described in the BSL Lesson 1.

The alignment step described in the previous subsection was also implemented for the EMG, but only after the computation of its linear envelope (as described in the next section).

#### 2.2.3 Electrodermal activity

For the EDA sensor, to avoid cross-talk between the two devices, BioPac's electrodes were placed on the index finger and BITalino's electrodes were attached to the ring finger. During the first 20 s, the subject is relaxed, and after this initial relaxation period, the subject answers ‘yes’ or ‘no’ to a series of questions.

Although we followed the procedure described in the BSL Lesson 9, translation and minor adjustments were made to the questions, in order to have them more adapted to the national context where the tests were performed. Examples of questions included in the questionnaire are translations of: ‘Are you a student?’, ‘Do you own a motorcycle?’ and ‘Have you ever visited another planet?’. The subject is instructed that he may answer truthfully or dishonestly, given that for our comparative analysis non-specific responses are sufficient.

For one subject, during EDA acquisition, the emission of BioPac pulses started before the BITalino was properly connected. This caused the first pulse to not be captured by the BITalino light sensor. Therefore, the second emitted pulse was the one used as the start of the activity.

#### 2.2.4 Electroencephalography

For the EEG, the ground electrode is placed behind the left ear. A bipolar measurement is performed, with two electrodes placed on the forehead, near positions Fp1 and Fp2 of the standard 10–20 system. Two 1 min activities are recorded: first, the subject is asked to keep his eyes opened, then he is asked to keep them closed. This follows the procedure described in the BSL Lesson 3.

A switching of electrodes happened during EEG acquisition, resulting in inverted signals between the two devices. Prior to segmentation, BITalino signals were therefore inverted.

### 2.3 Feature extraction

When the signal is very rich in high-frequency components, and considering that each device has an independent analogue-to-digital converter, it is virtually impossible to ensure that the sampling occurs at the exact same instant for both devices. This introduces a problem related to the fact that a very small offset in the sampling time can translate in significantly different measured quantities (as is the case for EMG data). Consequently, comparing raw EMG acquired by the BITalino and the BioPac does not provide the fairest comparison base. Similarly, EEG data, although not as rich in high components, varies very rapidly, and therefore comparing it in its raw form can be a challenge. Following the method presented in [10], we chose to compare these signals based on commonly extracted features, after applying common signal processing techniques.

For the EMG, the linear envelope of the signals was computed. First, a fourth-order forward–backward band-pass Butterworth filter with cut-off frequencies of 10 and 450 Hz was applied. The signals were then rectified before filtering with a fourth-order forward–backward low-pass Butterworth filter with cut-off frequency of 4 Hz.

EEG data is often analysed in terms of its frequency content. For each segment, an estimation of the power spectral density (PSD) was computed using Welch's method. Each segment was split into segments of length 1 s with overlap of 999 samples and a Hanning window was applied to them. The periodograms were then computed using a zero-padded fast Fourier transform of length 2048 samples. Finally, the individual periodograms were averaged.

Regarding the ECG, although a morphological raw comparison was undertaken, a simple processing step was also attempted. When using pattern recognition methods to analyse ECG records, be it for diagnostics or biometrics purposes, it is often beneficial to segment them and extract relevant features. A common segmentation procedure is to first identify the *R* peaks (corresponding to ventricular depolarisation), and then use only a predefined window around each peak. This method was also employed in our study, to allow a beat-by-beat comparison between the two devices. A window of 600 ms was used (200 ms before and 400 ms after the *R* peak). Before beat segmentation, ECGs were filtered in a more specific manner. First, two median filters with local window sizes of 0.2 and 0.6 s were applied to the signal. By subtracting from the original signal the result of this filtering, we were able to remove the baseline wandering. Then, the signal was filtered using a low-pass finite impulse response filter with a 40 Hz cut-off frequency. The filter used had an order of 300 and a flattop window.

### 2.4 Comparison metrics

The root mean squared error (RMSE) (1) can be used to measure the difference between the values recorded by the two devices (*x* and *y*, with length *n*). Since it is a scale-dependent metric, a normalisation step must be performed prior to its computation (2), where $\mu $ represents the data mean

(1) $${\rm RMSE}\lpar x\comma \; y\rpar = \sqrt {\displaystyle{{\sum\nolimits_{k = 1}^n {{\lpar x_{{\rm norm}}\lsqb k\rsqb - y_{{\rm norm}}\lsqb k\rsqb \rpar }^2} } \over n}} \eqno\lpar 1\rpar $$

(2) $$x_{{\rm norm}}\lsqb k\rsqb = \displaystyle{{x\lsqb k\rsqb - \mu _x} \over {\max x - \min x}}\eqno\lpar 2\rpar $$

A second metric, the coefficient of determination ($R^2$), was also computed. This value is a measure of the linear dependence between data from the two devices. More specifically, if we consider the least square fit between data from BioPac and BITalino, $R^2$ can be computed as shown in (6), considering the sum of squares detailed in (3), (4) and (5)

(3) $$SS_{xx} = \sum\limits_{k = 1}^n x\lsqb k\rsqb ^2 - n\mu _x^2 \eqno\lpar 3\rpar $$

(4) $$SS_{yy} = \sum\limits_{k = 1}^n y\lsqb k\rsqb ^2 - n\mu _y^2 \eqno\lpar 4\rpar $$

(5) $$SS_{xy} = \sum\limits_{k = 1}^n x\lsqb k\rsqb y\lsqb k\rsqb - n\mu _x\mu _y\eqno\lpar 5\rpar $$

(6) $$R^2 = \displaystyle{{SS_{xy}^2 } \over {SS_{xx}SS_{yy}}}\eqno\lpar 6\rpar $$

## 3 Results and discussion

Following the protocol described in the previous section, data was acquired for seven subjects and then segmented. The total number of segments, analysis time and number of events for each sensor are reported in Table 2.

**Table 2:** Total number of segments, analysis time and number of events for each sensor

Sensor	Number of segments	Total analysis time, s	Number of events
ECG	28	700	898 beats
EMG	14	394	154 muscle contractions
EDA	7	456	—
EEG	14	340	14 PSD estimations

The first comparison presented here is a simple morphological comparison. EDA, ECG and EEG data are compared using this approach. For EMG, EEG and ECG, a comparison based on common features is undertaken.

### 3.1 Morphological comparison

**Fig. 4:**
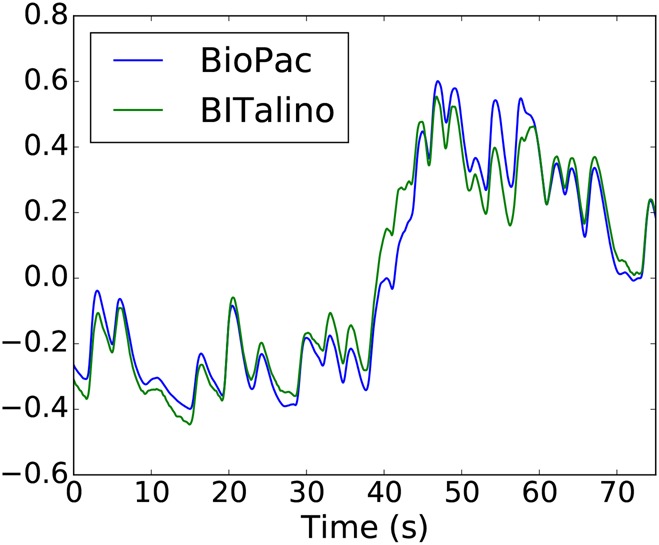
BioPac and BITalino EDA data from one subject, after signal alignment and filtering. Segments were scaled for visualisation purposes

RMSE and $R^2$ were computed to quantify the morphological differences between devices for EDA, ECG and EEG data. An example of EDA data acquired by the two devices is shown in Fig. 4, after signal alignment and filtering. The results of the comparison for these three sensors are summarised in Table 3, along with the remaining results presented in the next sections.

**Table 3:** RMSE and $R^2$ for all comparisons carried out

Sensor	Comparison type	RMSE	$R^2$
EDA	morphological	0.059 ± 0.029	0.948 ± 0.059
ECG	morphological	0.054 ± 0.012	0.830 ± 0.054
ECG	beat-by-beat	0.049 ± 0.016	0.914 ± 0.046
EMG	envelope	0.026 ± 0.009	0.989 ± 0.004
EEG	morphological	0.055 ± 0.012	0.693 ± 0.067
EEG	PSD	0.013 ± 0.005	0.968 ± 0.014

The RMSE is similar and low for all three signals. The $R^2$ is close to 1 for the EDA, indicating a high correlation between data acquired by the two devices. A lower value is obtained for the ECG, although still showing a strong correlation. Regarding the EEG, the decrease in $R^2$, which is still at around 0.7, can likely be attributed to the fast variations of this signal.

### 3.2 ECG beat-by-beat comparison

ECG segments were segmented beat-by-beat, as explained in the previous section. A total of 898 cardiac cycle waveforms were compared, resulting in a RMSE of 0.049 ± 0.016 and a $R^2$ of 0.914 ± 0.046.

For both the RMSE and the $R^2$, there is an improvement in comparison with the results obtained in the previous section. We can point out two main reasons for this. First, since portions of the signals are being left out, disparities between devices happening in these portions are minimised. However, since the main complexes are within the chosen window (P wave, QRS complex and T wave), the portions of the signal that are left out of this comparison have little relevance in what concerns the typical interpretation steps. The second reason stems from the alignment between the signals. Despite the methodology followed to ensure the initial alignment of the signals, slight differences in the device's internal clocks may cause the signals to drift apart from each other. This misalignment is more pronounced as time goes by. By using a small window and centring the beats by the *R* peaks, we are diminishing such disparities.

### 3.3 EMG envelope comparison

**Fig. 5:**
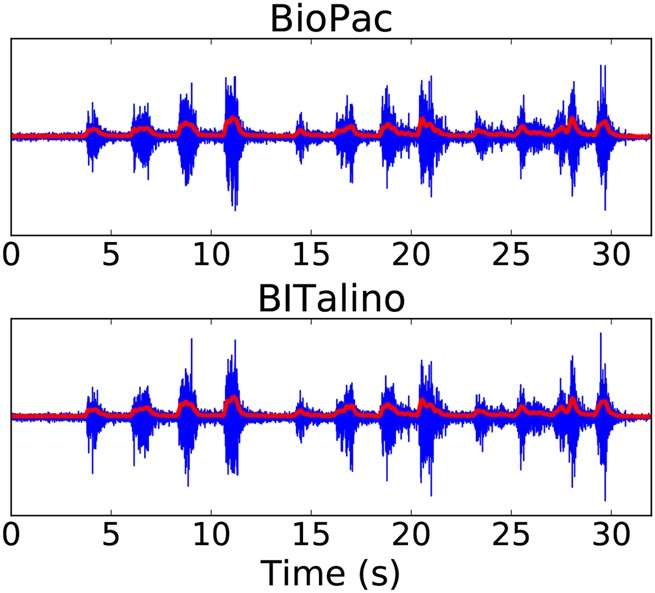
EMG data (in blue) and computed linear envelope (in red) for one activity

For each subject and each activity, EMG linear envelopes were computed. An example is shown in Fig. 5. The comparison between BITalino and BioPac data gives a RMSE of 0.026 ± 0.009 and a $R^2$ of 0.989 ± 0.004.

The low RMSE and high $R^2$ indicate a very close similarity between the linear envelopes computed for the two devices (Table 3). That is, although the high-frequency content of the signals makes it hard to morphologically match EMGs from different recorders, a simple and very useful processing technique confirms that the acquired signals are very similar concerning their information content.

### 3.4 EEG power spectrum comparison

Due to the sensitive nature of EEG acquisition, a preliminary test was made to assess the noise characteristics of the BITalino sensor. By placing the sensor in an input-referred configuration, we observed a peak-to-peak amplitude of 4.27 and a standard deviation of 0.43. Considering that the range of the sensor is $ \pm 41.24\, {\rm \mu V}$, this peak-to-peak noise amplitude corresponds to 5% of the full scale. Although this input noise can be sufficient to measure EEG, some sensitive applications may need to guarantee a higher SNR.

**Fig. 6:**
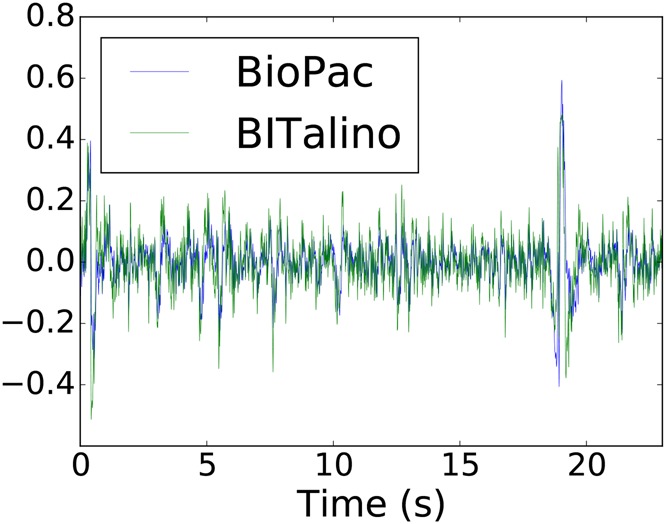
BioPac and BITalino EEG data from one subject, after signal filtering. Segments were scaled for visualisation purposes

**Fig. 7:**
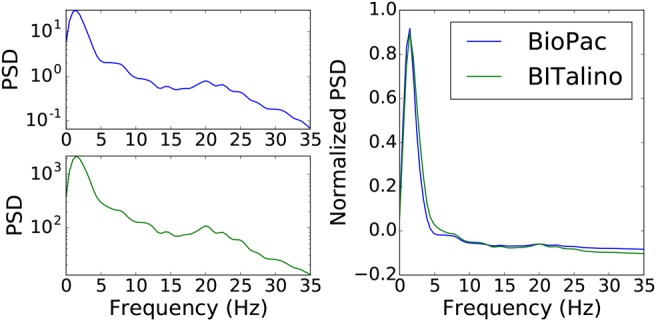
PSD (left) and scaled PSD (right) for one activity

An example of EEG data acquired by the two devices is shown in Fig. 6. The EEG PSD was estimated for each segment, as shown in Fig. 7, where the normalisation only takes into account values ranging from 0 to 35 Hz. Comparing the two devices gives an overall RMSE of 0.013 ± 0.005 and a $R^2$ of 0.968 ± 0.014. A comparison by EEG bands is presented in Table 4.

**Table 4:** RMSE and $R^2$ for different EEG bands

EEG frequency bands	RMSE	$R^2$
delta – 0 to 4 Hz	0.113 ± 0.029	0.857 ± 0.077
theta – 4 to 8 Hz	0.079 ± 0.04	0.995 ± 0.012
alpha – 8 to 14 Hz	0.027 ± 0.016	0.992 ± 0.009
beta – 14 to 500 Hz	0.003 ± 0.002	0.997 ± 0.001

For all bands, the RMSE is low and a high correlation between devices is obtained. We can note that the lowest band, delta, is the one where differences between devices are more noticeable. Overall, and comparing with the morphological results, it is clear that by applying this common processing technique the similarity between signals becomes more apparent.

## 4 Conclusions

With the goal of comparing data acquired by the BITalino against a reference equipment, we collected data simultaneously with the two devices. We followed a replicable data acquisition protocol for each one of the four physiological sensors (ECG, EMG, EDA, and EEG), and acquired data from multiple subjects. Pre-processing steps, including filtering and signal alignment, were implemented to ensure that the data could be fairly compared.

Morphological comparison of EDA and ECG signals showed a high similarity between data acquired by the two devices. For the EMG, the computation of the linear envelope resulted in a small difference and high correlation between the BITalino and the reference device. Regarding the EEG, spectral comparison showed a higher level of similarity between devices than the morphological comparison. Although the obtained results seem to be promising for benchmarking BITalino's EEG, the $ \sim 4\, {\rm \mu V}$ peak-to-peak noise floor can be sufficient to measure EEG, but will not always be. Due to the sensitive and specialised nature of this modality, we intend to deepen this analysis using a more appropriate EEG protocol (e.g. visual, auditory or other evoked potentials), following a different protocol than the one proposed in the BioPac lessons.

The empirical validation of BITalino against a reference device, as presented here, is important for educators and researchers working on many different applications. Since signals acquired with this low-cost and easy-to-use toolkit are similar to those acquired with well-established devices, it is expected that many more experiments can be carried out, leading to a faster development of different biomedical research topics.

## 5 Funding and declaration of interests

This work was partially supported by the Portuguese Foundation for Science and Technology, under grant no. PTDC/EEI-SII/7092/2014 and by the IT – Instituto de Telecomunicações under grant no. ‘SmartHeart’. Conflict of interests: None declared.
